# Minor and Unsystematic Cortical Topographic Changes of Attention Correlates between Modalities

**DOI:** 10.1371/journal.pone.0015022

**Published:** 2010-12-17

**Authors:** Luis F. H. Basile, Mirna D. Lozano, Milkes Y. Alvarenga, José F. Pereira, Sérgio Machado, Bruna Velasques, Pedro Ribeiro, Roberto Piedade, Renato Anghinah, Gennady Knyazev, Renato T. Ramos

**Affiliations:** 1 Laboratory of Psychophysiology, Faculdade da Saúde, Universidade Metodista de São Paulo, São Paulo, Brazil; 2 Division of Neurosurgery, University of São Paulo Medical School, São Paulo, Brazil; 3 Department of Psychiatry, Federal University of Rio de Janeiro, Rio de Janeiro, Brazil; 4 Department of Psychiatry, University of São Paulo Medical School, São Paulo, Brazil; 5 Department of Neurology, University of São Paulo Medical School, São Paulo, Brazil; 6 Universidade São Judas Tadeu, São Paulo, Brazil; 7 Institute of Physiology, Siberian Branch of the Russian Academy of Medical Sciences, Novosibirsk, Russian Federation; University of Queensland, Australia

## Abstract

In this study we analyzed the topography of induced cortical oscillations in 20 healthy individuals performing simple attention tasks. We were interested in qualitatively replicating our recent findings on the localization of attention-induced beta bands during a visual task [1], and verifying whether significant topographic changes would follow the change of attention to the auditory modality. We computed corrected latency averaging of each induced frequency bands, and modeled their generators by current density reconstruction with Lp-norm minimization. We quantified topographic similarity between conditions by an analysis of correlations, whereas the inter-modality significant differences in attention correlates were illustrated in each individual case. We replicated the qualitative result of highly idiosyncratic topography of attention-related activity to individuals, manifested both in the beta bands, and previously studied slow potential distributions [2]. Visual inspection of both scalp potentials and distribution of cortical currents showed minor changes in attention-related bands with respect to modality, as compared to the theta and delta bands, known to be major contributors to the sensory-related potentials. Quantitative results agreed with visual inspection, supporting to the conclusion that attention-related activity does not change much between modalities, and whatever individual changes do occur, they are not systematic in cortical localization across subjects. We discuss our results, combined with results from other studies that present individual data, with respect to the function of cortical association areas.

## Introduction

Over the last decades, a major scientific effort has been made in the attempt to map functions of cortical association areas. We have used as a basis for such possible mapping, the neuroanatomy of cortico-cortical connections, as opposed to the tradition of using purely psychological or behavioral distinctions. The relatively specific connections between visual areas [3], and other neocortical areas, particularly prefrontal [4]–[6] guided our task design, in the search for (prefrontal) cortical specializations of function, particularly of selective attention to different visual domains [e.g., 7–9]. However, in the last years we have been providing evidence for high individual variability, in the *sets of cortical association areas* active during non sensory-motor tasks: that is, the very concept that particular non sensory-motor functions are associated to the same anatomic areas in all individuals must be revised. This individual variability was firstly observed with respect to scalp and generator topography of slow potentials, classical correlates of attention, in relatively complex tasks [9], [10]. But only by analyzing Slow Potentials (SPs) from a simpler visual attention task we began to consider that this variability may be inherent to cortical physiology [2]. The variability was not observed for stimulation-related activity, in an explicit comparison between SPs and the visual N200s from the same data set. In a more recent study, we analyzed the topography of induced beta oscillations during the same simple task, as new correlates of attention, by corrected latency averaging of band-pass filtered epochs: this method allows the visualization of actual average voltage distributions that are not time-locked to stimuli [1]. In this study of beta oscillations, we observed that the topography of both baseline (pre-stimulus) oscillations, which increase in amplitude during the inter-stimulus interval (S1–S2 paradigm), as well as secondary, task-exclusive components, were also highly variable to individuals with respect to scalp topography and their generating sets of cortical areas. In this same study, the topography of frequency bands more closely related to sensory stimulation such as theta, proved to be much closer in topography across subjects (as expected, given the closeness in topography with evoked potentials), in a quantitative comparison with attention-related beta activity. We have started to reinterpret our first studies on slow potentials, on tasks involving explicit memorization, stimulus comparisons, categorization and feedback anticipation, where the same multifocal, complex, highly variable sources of slow potentials were always observed.

In the present study we further explore this hypothesis of individual-specific distributions of non-sensory-motor cortical activity, using the same visual task as in our last studies (where we analyzed SPs and first analyzed induced beta bands, [1], [2], [11]), for a dual purpose: 1) to qualitatively replicate those first beta localization findings on a slightly larger sample, and mainly 2) to allow an *individually-based* comparison with an auditory attention task. The main current interest was to observe how different would be the topography of attention correlates after displacement of attention from the visual to the auditory modality. We used as a reference for comparison the more purely stimulation-related frequency bands (theta and delta; along with alpha, [12]–[16]). In order to support the comparisons made by visual inspection, we computed the correlations, *within individuals*, *between* the cortical current distributions obtained for *the two modalities*, for baseline and main task-related activity, in all frequency bands. Those correlations were then transformed into Fisher's Z scores for *inter-individual* analysis, which we restricted to attention and stimulation representative bands (beta1 and theta), or to a combination of bands. Since we were also interested in the explicit computation of the significant part of the topographic *difference* between modalities, we complemented the analysis of correlations, by illustrating the significant part of each individual's difference of attention correlates between modalities.

## Methods

### Subjects

Twenty healthy individuals with normal vision and hearing, 12 male and 8 female, participated in the study. They ranged in age between 20 and 45 years, with no history of drug or alcohol abuse, and no current drug treatment. All subjects signed consent forms approved by the Ethics Committee of the University (Comitê de Ética em Pesquisa da Universidade Metodista de São Paulo).

### Stimuli and Task

A commercial computer program (Stim, Neurosoft Inc.) controlled all aspects of the tasks. Visual stimuli composing the cue-target pairs (S1–S2) consisted in small rectangles (eccentricity ±0.8°, S1: 100 ms duration, S2: 17 ms; white background). In half of the trials, the S2 rectangle contained a grey circle – the task target - with ±0.3° of eccentricity. A masking stimulus had the same grey level as the target (a ‘checkerboard’ grey and white square composed by one-by-one pixel size squares), and was continuously present, along with the fixation point, except during S1 and S2 presentation. S1 was followed by S2, with onsets *separated in time* by 1.6 seconds. The ITI was variable, ranging from 2.3 to 5 seconds. We instructed the subjects that a rectangle would be presented to indicate that 1.6 seconds later it would flash again but quickly, containing or not the target circle. The subject decided whether there was a target inside the S2 rectangle, and indicated presence of the target by pressing the right button with the right thumb or absence of the target by pressing the left button with the left thumb. We explicitly deemphasized reaction time in the instructions and measured performance by the percent correct trials (and their derivate false positives and hits), from the total of 96 trials comprising each task. An eye fixation dot was continually present on the center of the screen, as well as a stimulus-masking background, to prevent after-images. The parameters of the second task were identical to the above (even with maintenance of the visual stimuli but without targets), except for the addition of auditory stimuli, analogous to the visual, and instructions to detect auditory targets and ignore the visual stimuli: We placed a pure tone as auditory S1 stimulus (1000 Hz, 60dB, 100 ms duration) as close as possible to the visual stimulus in time (given the stimulation program limitations, there was a 100 ms delay between visual and auditory stimulus onset, but all subjects reported simultaneity when asked), and an identical auditory S2, except that 50% of them ‘contained’ the targets (defined by a slight, transient intensity reduction −10% for about 10 ms –– of the pure tone waveform).

### EEG Recording and acquisition of MRIs

We used a fast Ag/AgCl electrode positioning system consisting of an extended 10–20 system, in a 128-channel montage (Quik-Cap, Neuromedical Supplies®), and an impedance-reducing gel which eliminated the need for skin abrasion (Quick-Gel, Neuromedical Supplies®). Impedances usually remained below 5 kOhms, and channels that did not reach those levels were eliminated from the analysis. To know the actual scalp sampling or distribution of electrodes in each individual with respect to the nervous system, we used a digitizer (Polhemus®) to record actual electrode positions with respect to each subject's fiduciary points: nasion and preauricular points. After co-registration with individual MRIs, the recorded coordinates were used for realistic 3D mapping onto MRI segmented skin models, and later used to set up the source reconstruction equations (distances between each electrode and each dipole supporting point). Two bipolar channels, out of the 124-channels in the montage were used for recording both horizontal (HEOG) and vertical electrooculograms (VEOG). Left mastoid served as reference only for data collection (common average reference was used for source modeling) and Afz was the ground. We used 128-channel DC amplifiers (Synamps, Neuroscan Inc.) for data collection and the Scan 4.3 software package (Neurosoft Inc.) for initial data processing (before computation of averages). The filter settings for acquisition were from DC to 30 Hz, and the digitization rate was 250 Hz. The EEG was collected continuously, and epochs for averaging spanned the interval from 900 ms before S1 to 400 ms after S2 presentation. Baseline was defined as the 300 ms preceding S1. Epoch elimination was performed visually for eye movements and muscle artifacts, and then automatic: visual inspection served to eliminate occasional transient electronic or head movement noise present in channels other than EOG; epochs containing signals in either HEOG or VEOG channels above +50 or below −50 µV were eliminated. In our montage, the VEOG detected, typically, blinks as deflections above 130 µV in the positive direction.

MRIs were obtained by a 1.5 Tesla GE machine, model Horizon LX. Image sets consisted in 124 T1-weighed saggital images of 256 by 256 pixels, spaced by 1.5 mm. Acquisition parameters were: standard echo sequence, 3D, fast SPGE, two excitations, RT = 6.6 ms, ET = 1.6 ms, flip angle of 15 degrees, F.O.V  = 26×26 cm. Total acquisition time was around 8 minutes.

### Frequency-Time analysis

After artifact rejection, the signal from each channel was spectrally analyzed by means of a Short Time Fourier Transform (STFT), to obtain frequency-time charts of the induced spectrum of the interval from 700 ms previous to S1, to 400 ms after S2. To obtain the induced power spectrum [17], the time-frequency decomposition was made for each electrode and each trial, from DC to 30 Hz, and the resulting charts were then averaged, both for each electrode and across electrodes. The decomposition was computed on the EEG tapered by a sliding Hamming window, 256 points in size for frequencies over 5 Hz, and 512 points between 2 and 5 Hz, with a temporal resolution of N/10 (N being the number of temporal points of the raw signal), and a frequency resolution of 4 bins per Hertz. Then, we normalized the average power for each electrode to obtain Z-scores of increments or decrements in each frequency bin with respect to the power in the same frequency during the baseline (<P_j_>  =  (P_j_ - µ_j_)/σ_j_; given P_j_  =  spectral power at each time point in electrode j, µ_j_ and σ_j_ are the mean and standard deviation, respectively, of the average power during the baseline for the electrode).

### Computation of corrected latency burst averages

We used corrected latency averaging instead of conventional event-related potential (ERP) averaging or event-related (de-)synchronization (ERS/ERD), because only in this case we would be able to analyze task-related potential distributions that are not time-locked to task-events. Conventional ERP averaging results in exclusively time-locked activity (e.g., induced beta cannot be observed), whereas ERS/ERD analysis allows visualization of ‘electrical power’ distribution but not of actual voltages (which would not allow our further current density reconstruction analysis). According to the observed induced frequency bands for each individual (and to results from our previous study, [1]), we chose the bands for band-pass filtering of the original artifact-free EEG epochs: Butterworth, 96dB rolloff, 0–1 Hz for SPs, 1–3 Hz for delta, 3–7 for theta, 7–9 for alpha1, 9–12 for alpha2, 18–23 for beta1, and 23–29 Hz for beta2). The resulting filtered epochs were then processed by an algorithm developed by ourselves for searching the peaks of bursts within the task-time windows of interest (a detailed schematic representation of the method is found in Figure 1 of [1]). Filtered epochs were thus cut again starting from positive voltage peaks, resulting in new epochs, ranging from 400 ms before to 400 ms after the peaks. A minimum of 60 epochs was the criterion for averaging, for each individual and frequency band, using each channel in the search for peaks (we included the typically few error trials, since our main interest was in the ISI, pre-S2 window). Then, a grand average was computed using the averages obtained by guidance from each channel. In all cases, we also computed pre-S1 burst averages (representing the baseline topography for each frequency band), where the program searched peaks from −400 to 0 ms before S1, for comparison with the task-induced bursts. We computed the total power of corrected latency average peaks for all bands, and the overall results were tabulated, after conversion of into SNR values. We also computed Spearman correlations between SNR in all bands and task performance.

**Figure 1:**
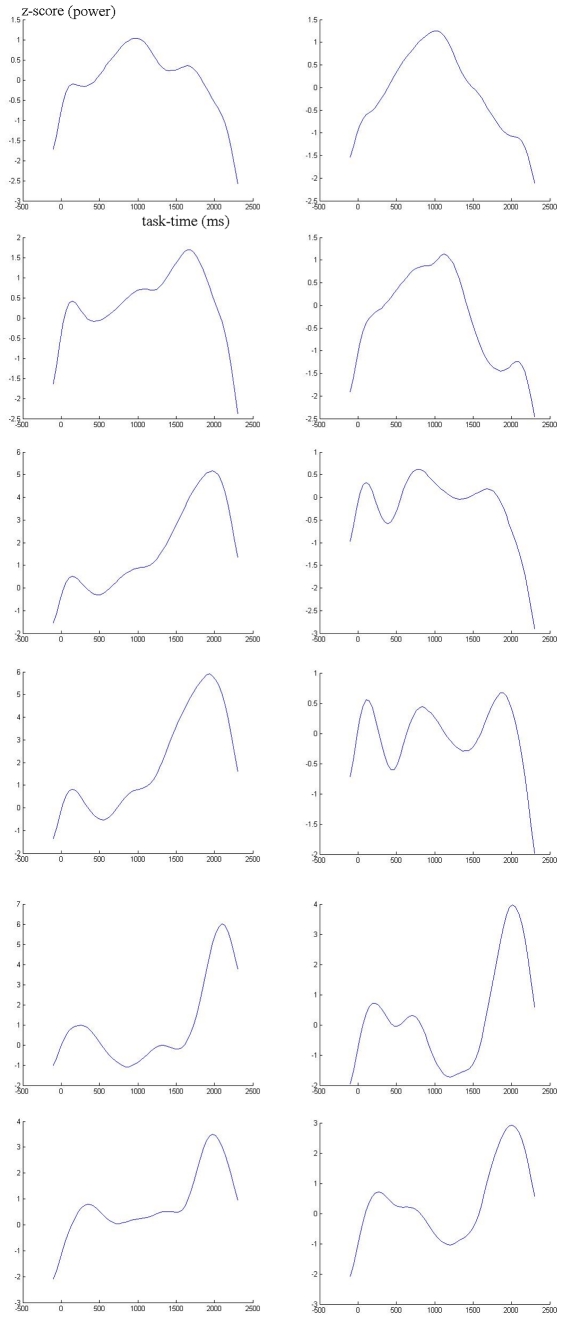
Task-related EEG Power. Time course of power (converted to z-score with respect to baseline) in one example subject. At left, visual task, right, auditory. From top to bottom, beta2, beta1, alpha2, alpha1, theta and delta.

For a systematic visual inspection, we computed realistic three-dimensional topographic maps of the scalp distribution of averages for each frequency band and its main ICA components, over the reconstructed scalp anatomy. To this purpose, we used a commercial sotfware (Curry V 4.6, Neurosoft Inc.), that co-registered individual MRI sets (skin model, see below) with the actual position of each electrode with respect to common landmarks, and linearly interpolated the instantaneous voltage values to obtain continuous maps.

### Intracranial source reconstruction

The computed averaged bursts, MRI sets and electrode position digitization files were the raw data for all further source analysis (Curry V 4.6, Neurosoft Inc.). A detailed description of the reconstruction procedure, and a discussion on the criteria for method choice and shortcomings, as well as on critical steps, may be found in the methods sections of previous publications [2], [10]. Noise in the data was defined as the variance of the 20% lowest amplitude points in each average. For the inclusion of a ‘noise component’ into the source model, the physical unit-free or ‘standardized’ data (with retained polarity) were decomposed by Independent Component Analysis (ICA), which searches for the highest possible statistical independence or redundancy reduction between components (in this case, space-time averaged data patterns), a robust method of blind signal decomposition/deconvolution (for a review, see e.g. [18]). ICA was applied to each individual's whole space-time data set, i.e., to the m × n data matrix (m used channels X 201 time samples corresponding to the 800 ms composing the averaged bursts). Finally, we fed the reconstruction algorithm with the main ICA component(s) as data to be fitted, with SNR>1. In practice, in all cases, only two or three space-time ICA components were then modeled. MRI sets were linearly interpolated to create 3-dimensional images, and semi-automatic algorithms based on pixel intensity bands served to reconstruct the various tissues of interest. A Boundary Element Model (BEM) of the head compartments was implemented, by triangulation of collections of points supported by the skin, skull and cerebrospinal fluid (internal skull) surfaces. Mean triangle edge lengths for the BEM surfaces were, respectively, 10, 9 and 7 mm. Fixed conductivities were attributed to the regions enclosed by those surfaces, respectively, 0.33, 0.0042 and 0.33 S/m. Finally, a reconstructed brain surface, with mean triangle side of 3 mm, served as the model for dipole positions, corresponding to a range from around 9 to 23 thousand points, depending on the head size. The electrode positions were projected onto the skin's surface following the normal lines to the skin. The detailed description of the assumptions and methods used by the “Curry 4.6” software for MRI processing and source reconstruction may be found elsewhere (e.g., [19]–[21]). The analysis program then calculated the lead field matrix that represents the coefficients of the set of equations which translate the data space (SNR values in the set of channels per time point) into the model space (the thousands of dipole supporting points). The source reconstruction method itself was Lp norm minimization, with p = 1.2 both for data and model terms. The regularization factor, or λ values to be used, typically converged after repeating the fitting process two to three times (λ gives the balance between goodness of fit and model size).

### Statistics of reconstruction results

In order to quantitatively compare *inter-individual* or group results between the visual and auditory conditions, we used *inter-modality correlations as the measure of similarity* between the identically ordered sets of dipoles obtained for (representative) chosen frequency bands or their combinations: 1) based on an analogous comparison from previous studies between stimulus versus attention correlates (N200 versus SPs, [2]; theta versus beta, [1]), we here chose the inter-modality correlations (their Fisher's Z transforms) of the second ICA components of theta versus beta1 bands: we chose theta as the main ‘stimulus-related’ band based on previous findings and current induced power analysis, since in most subjects its distribution is virtually identical to the visual N200 or auditory N100 topographies, whereas beta 1, representing ‘attention-related’ activity, was observed to be stronger than beta 2, more uniform in peak frequency across modalities); and then 2) we also used the average of the Fisher's Z transforms of correlations in theta combined with delta versus the transforms of correlations in beta1 combined with beta2 and with SPs (pooled sets of bands mainly related to stimulation or attention). Complementarily, to evaluate the *intra-individual differences* in cortical topography of attention correlates between modalities, we computed a point-by-point dipole strength difference vector. First, each data pair to be compared was adjusted or ‘normalized’ to became of equal total strength or mean (the weaker set of dipoles was multiplied by a scalar number so that its average would match the stronger set; this method is analogous to the scaling of voltages to a common global mean field power, to emphasize topographic differences and deemphasize mere differences in amplitude, which proved useful in a inter-individual comparison in a clinical application; [22]). Second, we transformed the difference vector distribution into z-score values. Finally, we considered the points of major contribution to condition differences as absolute individual Z values, corresponding to global Z  = 2.57, which depending on the individual's number of point-to-point comparisons (i.e., the number of dipole supporting points, in proportion to head size), ranged from Z  = 3.48 to Z  = 3.68. The cortical distribution of such points of Z-score beyond the individual's threshold was thus known *a posteriori*, by using the corresponding current density cutoff value as the minimum current to be plotted. Finally, we also checked current distributions for a very low cutoff value of Z  = 1.96.

## Results

### Task Performance

All subjects reported that performance was relatively easy, provided that they were strongly attending during the critical time of S2 presentation. The overall average performance in the visual task was 83.2% correct responses (standard deviation 15.1%) and 89.6% in the auditory task (standard deviation 11.7%). Neither this difference was statistically significant (t-test, p = 0.08), nor for false positives (p = 0.73) or correct hits (p = 0.21).

### Topography by visual inspection and Correlations of source reconstruction results

**Figure 2:**
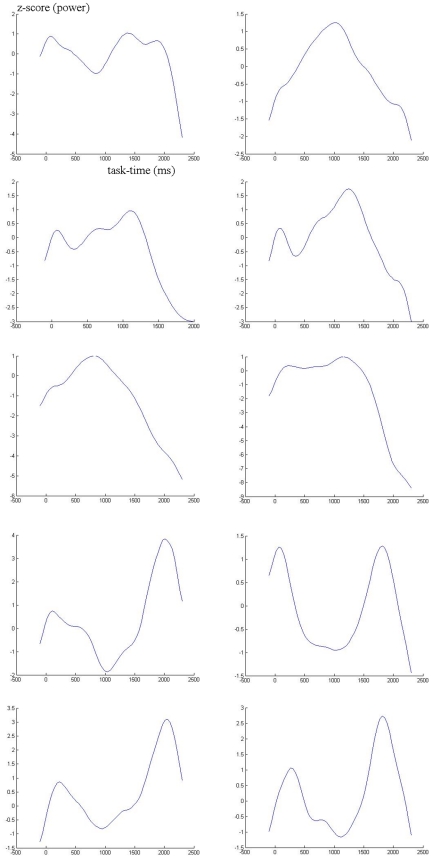
Task-related EEG Power. Same as figure 1, but in example subject presenting a single alpha band.

**Figure 3:**
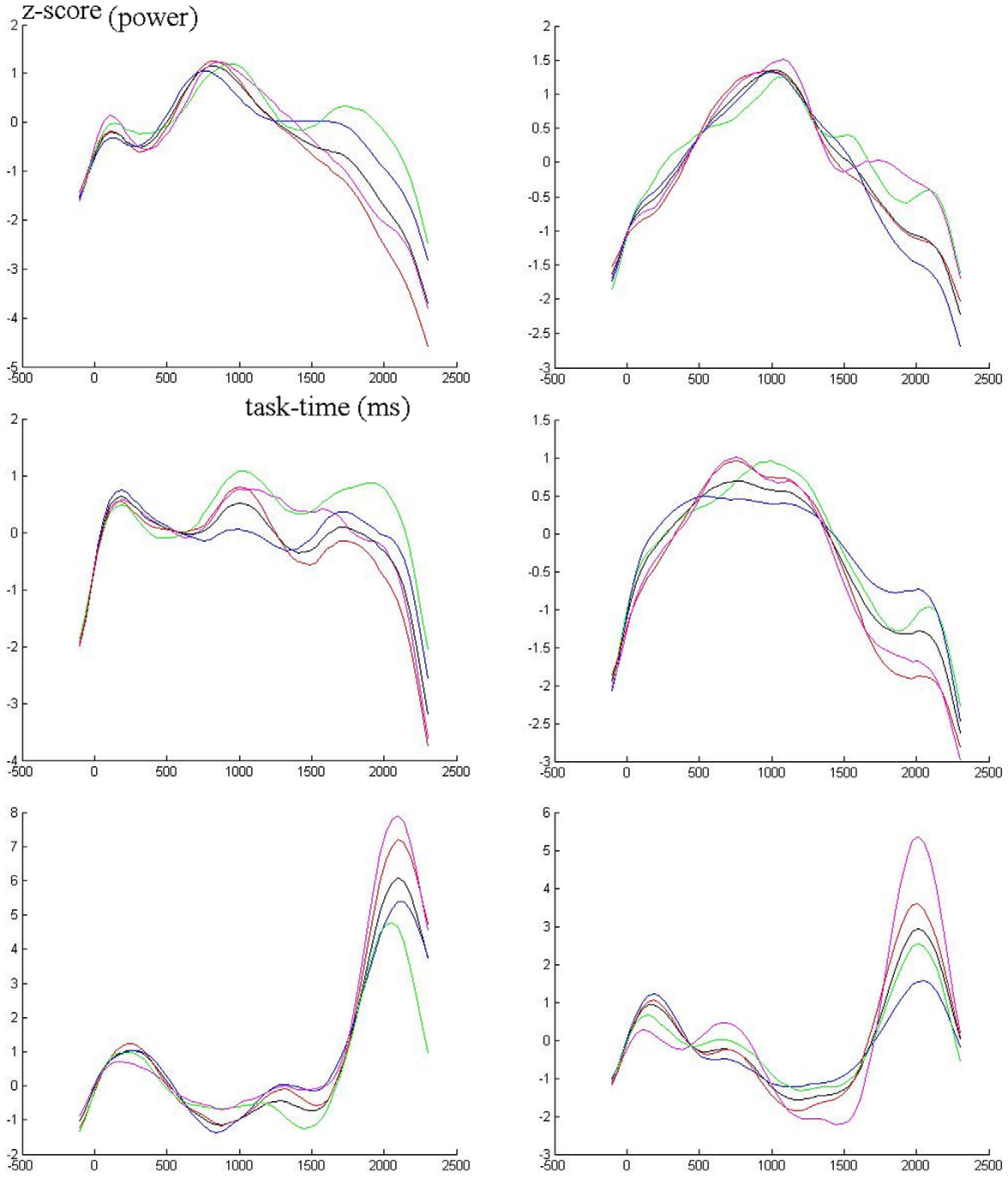
Task-related EEG Power. Time course in three bands (top to bottom: beta2, beta1 and theta) separated by electrode groups, representing scalp regions: black = all electrodes; green = temporal; red = parietal; magenta = occipital; blue = frontal.

Table 1 shows the baseline and task-related root mean global field power changes, converted to SNR values, for both tasks, averaged across subjects. Significant task-related power changes (always increases) are indicated in the bands where it occurs. Notice the lack of increases in the theta band (and alpha1 in auditory task), which as in previous studies, mainly concentrates power (phase-locking to stimuli) in the immediate post-stimulus windows, peaking close to the N200. Only the alpha2 baseline power during the auditory task correlated significantly with performance in the same task (negatively; −0,563, p = 0.01). Since corrected latency averages partially collapse task-time course information, leading do independent components mainly separated by phase, we exemplify task-related electrical power changes in two subjects in Figures 1 and 2, from delta to beta 2 bands, and the similar contributions from separate groups of electrodes to three bands, by scalp region, in one subject (figure 3). Visual inspection of topographic isopotential maps and source reconstruction results supported a qualitative replication of our main recent findings: 1- Scalp topographic characteristics and the corresponding current density distributions were variable across subjects, for first ICA components, and especially so for second or task-related components, in both visual and auditory conditions; 2- the baseline activity voltage distribution was again identical to the first ICA component during the ISI, and similar across all frequency bands for all subjects (but typically a little more complex – less smooth isopotential lines - in the beta bands), with three exceptions, all of which also previously observed: a) the task-related pattern in the delta band was stronger (ranked as first ICA component instead of second) than the baseline pattern in 10 subjects; b) a different, peculiar alpha2 pattern was stronger than the one common across frequencies in three subjects (the remaining subjects also had a resting or baseline alpha proper pattern, but secondary to the baseline pattern common to other frequencies); and c) slow potentials are purely task-related, having no topographic similarity with all other frequencies.

**Table 1:** Grand average of root mean global field power (computed at peak of corrected latency averages) converted into SNR, by frequency band (V = visual task; A = auditory task).

SNR	baseline		task	
	V	A	V	A
Beta 2	5.60	5.67	7.21**	6.83**
Beta 1	6.54	6.66	7.72**	7.50**
Alpha 2	5.18	5.15	6.19**	5.80**
Alpha 1	4.93	5.57	5.94**	6.04
Theta	5.76	5.50	5.36	5.65
Delta	3.32	3.15	8.89**	6.43**

Significant increases in power with respect to baseline are indicated by asterisks (p<0.01, paired t-tests).

Regarding the results of our main present interest, similarity or difference between secondary components in auditory versus visual conditions in chosen frequency bands, visual inspection was compatible with our expected results: 1- first ICA component topographic patterns were virtually indistinguishable between conditions, in all including the beta bands. We count the baseline beta activity among the attention-related components, since it systematically increased in power from pre-S1 activity both in the previous and present study (by 39% in the present study), as opposed to theta and delta baseline components, that even decrease in amplitude in some subjects; 2- secondary component patterns of stimulus-related bands, theta and delta, were clearly different between conditions. But the most interesting result was the 3- inter-modal similarity between task-related or secondary beta components.

**Figure 4:**
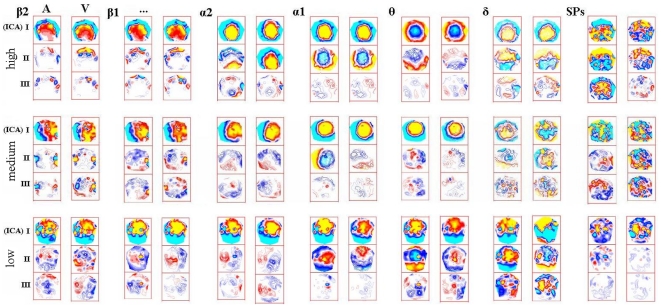
Audio-visual topographic comparisons. Examples of topographic maps (three-map sets represents first or baseline (I), second (‘task-exclusive’ used for comparisons- II) and third (III) ICA components in each frequency band (indicated at left of each auditory = A visual = V pair; nasion on top of maps). Each of the three line sets is labeled according to each representative subject (“high”, subject that presented the highest inter-modality correlations of attention-related results; “medium”, subject of median correlations; “low”, subject of lowest correlations in the group). Hot versus cold colors indicate opposite polarity of purely spatial patterns (temporal patterns were complex, always with phase differences between components).

**Figure 5:**
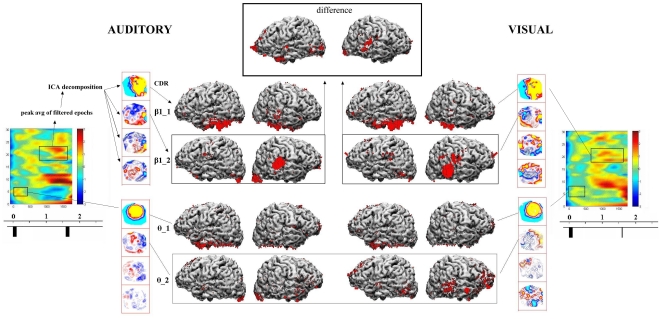
Audio-visual cortical generators comparisons. Example of source reconstruction results, for which further analyses were performed (correlations for similarity and significant differences for distinction between auditory and visual activity), in the subject with median inter-modality correlations. At extreme of both sides, z-score transformed frequency-time plots of task-related power changes (bars below represent stimuli). Processing steps indicated for the auditory beta1 (peak averaging of band-pass filtered epochs, ICA decomposition of resulting averages, and Current Density Reconstruction – CDR - of single ICA filtered components – only first and second: e.g., β1_1 = main or resting beta1 component; β1_2 = second or task-related beta1 component). CDR results for the second ICA components were used both to compute significant audio-visual differences (example for β1 in this subject on top of the figure) and correlations between those tasks. Bottom half of CDR results exemplify results for the theta band.

Figure 4 shows the topographic patterns obtained in three example individuals, selected for representing the lowest, median and highest audio-visual topographic correlations in the attention-related bands. It may be noticed that in all cases the first components are similar across bands, with increased topographic complexity in the beta bands. One may also notice the overall similarity between secondary beta components, in spite of the difficulty in simultaneously controlling the number of isopotential lines to illustrate component maps, due to the much stronger first components. Major differences can be seen particularly between auditory and visual theta band secondary components. Figure 5 shows the source reconstruction results (along with main steps of analysis) in the example subject of median audio-visual correlations in the attention-related bands. The thin rectangles depict the results for secondary or ‘task-exclusive’ components in the main bands for which inter-modal correlations were compared, theta and beta1.

**Figure 6:**
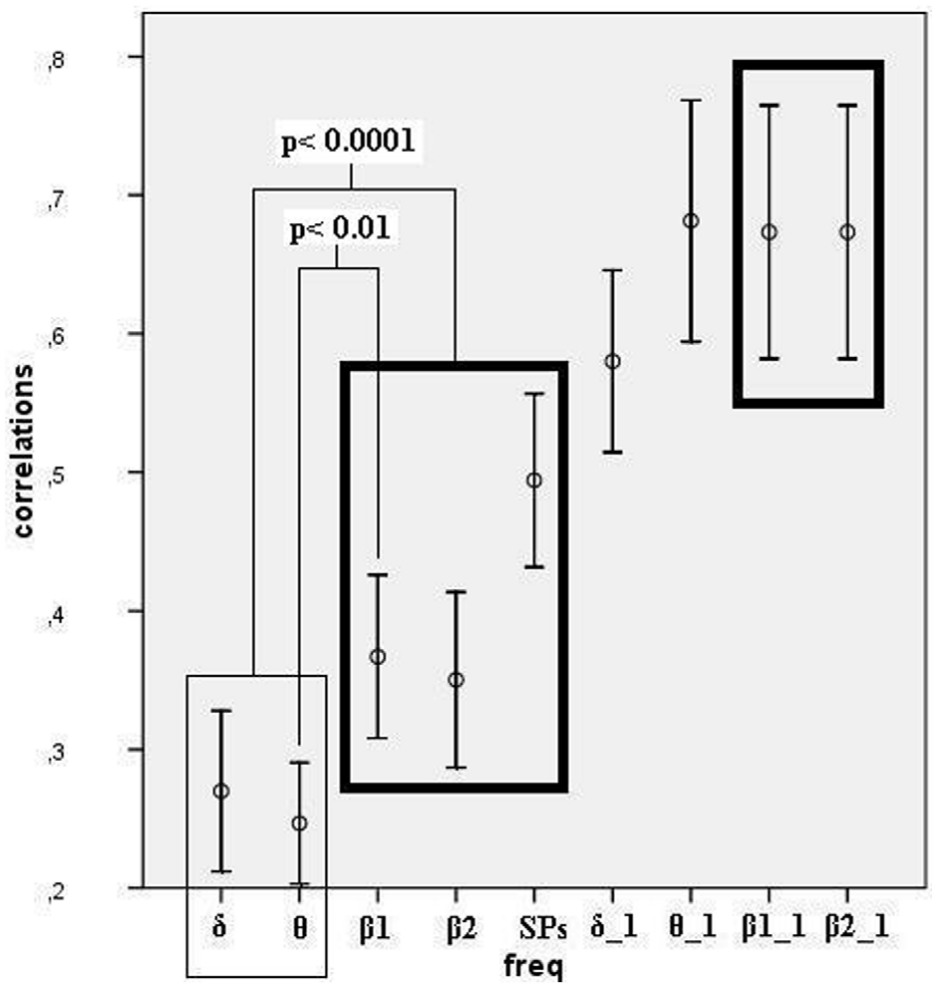
Audio-visual correlations. The similarity in current density distributions between visual and auditory tasks was measured by the correlations between the identically ordered sets of dipole strengths, in each frequency band and ICA component (Inter-subject averages are shown only for illustration; Comparisons were performed between Fisher's Z transformed correlations; first components: “band”_1; secondary components: w/o extension; bars: 95% confidence interval of the mean). Thick rectangles depict attention-related bands, and thin rectangle, stimulus-related bands. However, first beta components were not used in comparison due to their trivial high correlations, common to most bands (see text).

Given the confirmed normal distribution of our data, we computed parametric correlations between audio and visual cortical current distributions, and they quantitatively supported the observations by visual inspection (figure 6 shows the average audio-visual topographic correlations across subjects in various bands). First, by considering an arbitrary cutoff value of Pearson's r  = 0.5, stimulus versus attention-related bands could be separated in the following way: at least one of the attention-related bands had their second ICA components with high significant audio-visual correlations in all but one subject, whereas in no case was there a high significant correlation between theta or delta task-related components. Second, the actual correlation values, according to visual inspection, were highest in all frequency bands for first ICA components, intermediary in value for Slow Potentials and secondary beta bands, and minimal for delta and theta bands.

### Inter-individual comparison between attention and stimulation-related audio-visual topographic correlations

**Figure 7:**
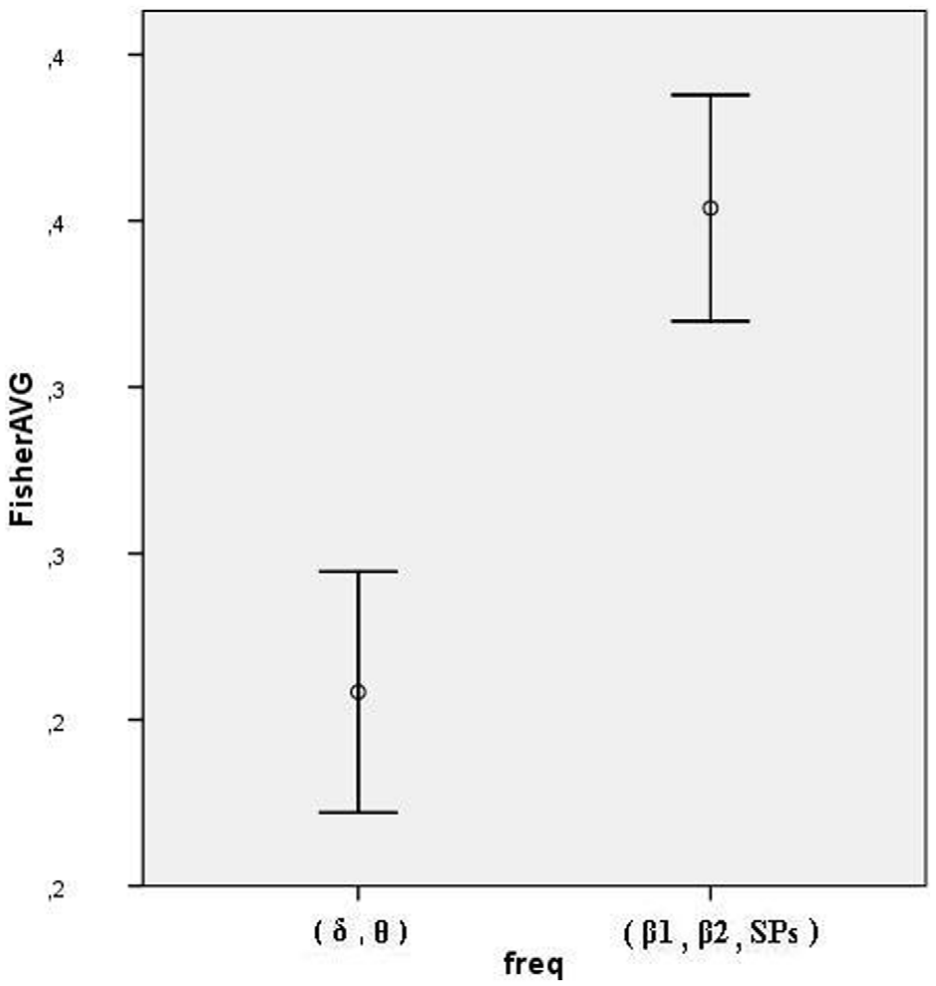
Audio-visual correlations. Pooled Fisher's Z averages, separating stimulus from attention-related activity.

After conversion of correlations between auditory and visual-related current density distributions into Fisher's Z transforms, we performed paired t-tests that resulted in highly significant differences, both in the comparison between the transformed correlations in theta and beta1 bands (t = −2.99, p<0.01), and between the Fisher's Z scores averaged across theta and delta bands, and the scores averaged across beta1, beta2 and slow potentials (t = −5.885, p<0.0001). Figure 7 illustrates the pooled Fisher's Z scores representing the stimulus and attention-related bands.

### Cortical distribution of significant contribution to within individual differences between visual and auditory attention correlates

**Figure 8:**
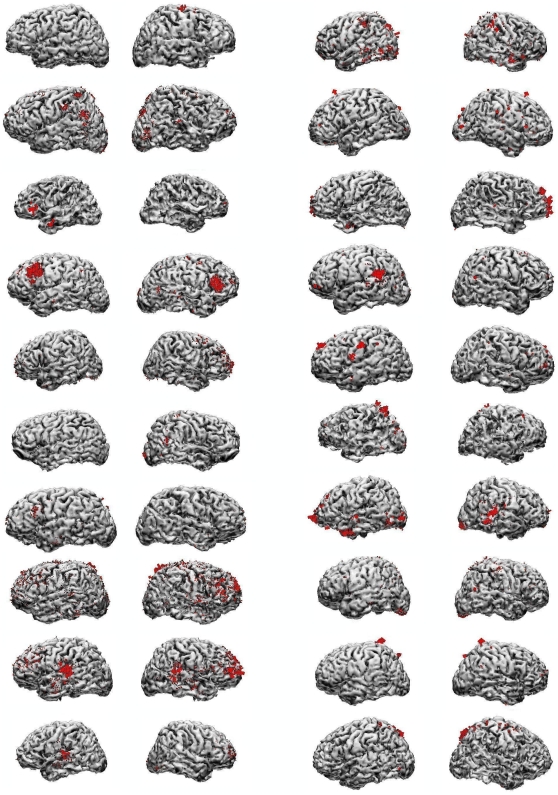
Auditory-visual attention differences. Significant part of difference in dipole strength distributions between auditory and visual tasks, in all subjects. Illustrated are the differential currents of absolute z-score value beyond a global z = 2.57, adjusted to number of dipole supporting points in each individual (range: z = 3.48 to z = 3.62). Relative local current density (to individual maximum) is represented by red arrows and proportional to arrow size (due to wide differences between maximum and minimum individual values, actual arrows can be noticed entering –or arrow heads exiting- the brain only in a few cases of high local current density, e.g., first and second subjects on top of right column).

The Z-score transformation of the point-by-point audio-visual differences in dipole strength distributions were computed for the task-exclusive beta1 and beta2 components, and for the SPs. In none of the three cases did any single cortical cytoarchitectonic area contain foci of (differential) current density common across subjects. Figure 8 shows the localization of the significant portion of differences in current distribution between tasks, in the beta1 band, in all subjects. The task differences for SPs were not localized in the same areas as beta1, but the two beta bands were typically very similar, as previously observed [1]. The inspection of current distributions after a very permissible cutoff value, of course increased the extent of current density foci, but in all cases (three bands) resulted in the same overall conclusion: not a single cytoarchitectonic area containing current foci was common to all subjects.

## Discussion

The present work reproduced previous findings of high inter-individual variability of cortical activity patterns during the visual attention task [1], [2]: varying sets of cortical areas and current density distribution explain the direct electrophysiological attention correlates, both induced beta bands and slow potentials across individuals (we left the alpha band out of the analysis on purpose, due to the complexity of relations with task events, variable set of sub-bands across subjects, and controversial functional interpretations, previously discussed in an analysis of the visual task, [1], [11]). The literature that presents individual data, mainly using functional magnetic resonance imaging (fMRI), corroborates our findings on high individual variability, whenever non sensory-motor tasks are studied, and consistently adverts against ‘brain averaging’ (e.g., [23]–[29]). Occasionally, this variability has been observed even in the case of passive (cutaneous) stimulation (e.g., [26], [27]). Those reports are in accordance with our own unpublished fMRI data from 60 individuals performing auditory and visual memory recognition tasks, where a high individual variability was seen, as opposed to their simpler, predictable and common patterns of activity following passive audiovisual stimulation. Taken together, all those observations challenge the validity of our search for predetermined functions of cortical association areas, of course depending on the definition of functions. We distinguish (expecting) attention proper from consequences of attention on stimulation and detection, whose correlates are the well known modulation of evoked and endogenous potentials, for instance. In our anatomy-based search of regional cortical specialization of function, we used the concept of function as the mentally dealing (e.g. comparing, associating) with different sensory materials within a modality, such as spatial versus non-spatial ‘mental operations’. Excepting the sensory-motor components, individuals seem to implement such common ‘functions’ using varying cortico-cortical circuits. On the other hand, putative functions abstracted exclusively from behavioral measures, may or may not correspond to distinct physiological correlates or implementation, at least in the present, macro-anatomical sense: different timing or accuracy of response may not necessarily correspond to implementation by differing (cortical) circuits.

A second qualitative replication was the observation of the topographic similarity of baseline activity between frequency bands in each individual, with the same exceptions of slow potentials, and a main alpha component in some subjects.

The new contribution was the explicit, *within subjects* comparison between the generators of correlates of the visual task, and those obtained when attention was displaced to additional auditory stimuli. Measures of similarity between correlates of both modalities, the point-by-point correlations between dipole strength distributions, had an overall correspondence with visual inspection. They were clearly higher for attention correlates (induced beta oscillations and SPs) than for more closely stimulus-related activity (theta and delta bands). Thus, even the secondary or ‘task-exclusive’ induced beta oscillations were significantly closer to each other in topography between modalities than theta and delta topographic patterns. The baseline beta1 and beta2 topography, corresponding to the main induced attention correlate due to its strength and increase during the ISI, is virtually indistinguishable between conditions, and present the highest inter-modality correlations. Those highest correlations correspond to practically indistinguishable patterns at visual inspection. However, they may be considered trivial, since they happen to the baseline components in all frequency bands (we have data showing pre-S1 baseline topography also to be indistinguishable from quiet resting), only in lesser degree in the beta band.

Our findings indicate that attention, at least in the broad sense that we use, is related to activity in distributed sets of cortical areas peculiar to each individual. Even in the case of the present major, modality change in attention, the known electrophysiological correlates do not appear to significantly change, suggesting an individual-specific, but modality unspecific cortico-cortical network. Given the high number of compared dipole strengths, on the order of 10^4^, we complemented our analysis of similarity by an explicit treatment of the intra-individual condition differences as well. We illustrated the loci of the portion of current distribution by a rigorous cutoff value of the z-transform of the difference vector between conditions (but also inspected results after a non-rigorous threshold), for the attention-related frequencies. We were interested in knowing whether any inter-individual systematic trend in anatomical localization occurred, however small the overall changes following the displacement of attention could be considered after the correlation analysis.

Once more, no common area across individuals showed to be specifically related to the shift of attention to the auditory modality, even when the low significance threshold was used. This occurred in the case of the bands that we consider more directly related to attention: the baseline components of beta1 and beta2 bands, that are enhanced and thus along with SPs form the major attention correlates, and the second ICA beta1 and beta2 components (“task-exclusive” since their topography is not present during baseline activity). This latter method may be considered analogous to ‘task-subtraction’, and more exclusively sensitive to the variable of interest, the displacement of attention, minimizing the effects of other factors that could interfere with inter-individual variability. For instance, although based on a still insufficient number of replication cases, we do suspect of a significant change even in the baseline topography in the course of years, as opposed to a few months. In any case, if confirmed, this instability would be one more ‘uncontrollable’ source of individual variability. Thus, idiosyncratic cortical activity seems to be a robust phenomenon, even when those other factors are considered, such as: task complexity and corresponding individual strategies of execution (whose minimization of variability was the main intent of the present task design, a simplification of Posner's task, [30], [31]; discussed in [2]); the unavoidable inclusion of some degree of memorization in any task; some extent of stimulus evaluation in any voluntary action; even the extreme possibility of a lack of relation between our measures and behavior [2], [9]; or a “methodological threshold” limitation [1]. Regarding the threshold problem, we may place our studies in one extreme, of a case-by-case description of occurrence of activity foci in estimated cytoarchitectonic areas, where the other extreme is represented by spatial grand averaging studies, which may occasionally present results that are not shared by all individuals. However, exactly due to the inconsistency in active areas between individuals, some groups of authors may be placed in an intermediary position between those extremes. They are developing interesting methods to account for such variability, with accompanying theoretical interpretations. One example is the application of the concept of ‘biological degeneracy’ to functional studies and Neuropsychology (‘many-to-one’ mapping of areas-funtion) and the ‘multisubject network’ method (e.g., [32], [33]), and ‘fuzzy clustering’ [34]. Another example is the data-driven delimitation of distributed “partially segregated networks of brain areas” to be re-approached to individual, fMRI and EEG data [35], [36]. Both example efforts represent a compromise between the pure presentation of individual data and grand spatial averaging. But we believe they cannot still replace the first extreme, since some methodological steps somewhat remove their final spatial results from actual physiological changes, such as the occasional stress in ‘regions of interest’ (all subjects included in an area, even when ‘extreme’ or ‘outliers’ in original measures), or statistical clustering of ICA extracted individual patterns and use of very low correlations between fMRI and EEG results.

But we must still contrast our conclusions with those from other psychophysiological studies presenting individual data, mainly those using fMRI. Most reports are concerned with intersubject variability, but in the extent or intensity of activation *in pre-chosen regions of interest* (e.g, [37], [38]). Studies explicitly showing the *variability in the actual sets and distribution of active areas* are less common [23]–[29]. Our findings, along with the last mentioned studies and our own unpublished fMRI results on visual and auditory recognition tasks versus mere stimulation, suggest that non sensory-motor cortical cytoarchitectonic areas cannot be expected to implement any pre-established physiological function, at least when function is conceived as classical, general psychological processes such as expecting attention, perception or target detection, conception, effort of memorization or evocation. Of course, among the infinitude of non-physiologically or descriptively-driven constructs of contemporary Psychology, if considered ‘functions’, may prove to be rather localized in cortical domains (as believed to be the case for ‘error detection’ or auditory ‘change-detection’, when considered as outside of the sensory domain). The commonplace observation from clinical practice in Neuropsychology, of variable symptomatology and degrees of impairment following lesions on common areas across subjects, corroborates this view. Most cases of functional claims regarding particular cortical areas, but based on group averages, neglect the fact that a percentage of subjects do not show any activation at all in those areas. Finally, it is worth mentioning once more (as discussed in [9]), that whatever the concept of function may be, an *unrestrained* literature search will lead to an enormous number of functional studies involving most cortical areas, and reciprocally, searches merely using any given cortical association area will result in functional hypotheses difficult to reconcile with each other, and especially so with a general biological theoretical framework.

A limitation of our studies is the restriction of observations to cortical activity. Circuits implementing whatever psychological process to be considered in the future as in fact basic or elementary, may essentially involve the interaction between individually variable functional cortico-striato-pallido-thalamo-cortical circuits (CSP ‘loops’) and invariable subcortical structures. Among the latter, a critical role must be ascribed to the structures mediating the interaction between the telencephalon and effector systems (somatomotor, vegetarive and endocrine), and between the telencephalon and the (aminergic) nuclei and cell fields of relatively diffuse ascending projections. Those structures are the extended amigdala, the lateral septum, and the habenulo-interpeduncular and mamillo-tegmental axes [39]. Thus, it may be the case that an absolutely universal part of circuits across subjects may only be found on the highly convergent interactions between CSPs and such mediating structures, which in principle are inaccessible to EEG/MEG and probably so to fMRI, given their minute proportions.

In sum, the patterns of cortical electrical activity observed even during relatively simple mental tasks appear to reflect the high inter-individual variation that we intuitively perceive in human associative thinking. Ever present and very general psychological facts in voluntary action such as ‘association’ or ‘problem-solving’, may one day prove to be the actual correlates of the topographically variable electrophysiological measures reported here. Whatever proves to be the case, individual-case mapping of task-related neurophysiological measures, independent from preconceived expectations, seems essential to both clinical and physiological understanding of human mental functioning.
